# Lipoxygenase and Xanthine Oxidase Inhibition and Antioxidant Potential of Fractions Obtained by Multistep Extraction of Artist’s Bracket (*Ganoderma applanatum* (Pers.) Pat.) and Red-Belted Bracket (*Fomitopsis pinicola* (Sw.) P. Karst.)

**DOI:** 10.3390/antiox15060663

**Published:** 2026-05-25

**Authors:** Michał Świeca, Agata Michalska, Katarzyna Lisiecka, Małgorzata Sierocka, Piotr Jarocki, Natalia Żurek, Ireneusz Kapusta

**Affiliations:** 1 Department of Biochemistry and Food Chemistry, University of Life Sciences, Skromna Str. 8, 20-704 Lublin, Poland; agata.michalska@up.edu.pl (A.M.); katarzyna.lisiecka@up.edu.pl (K.L.); malgorzata.sierocka@up.edu.pl (M.S.); 2 Department of Biotechnology, Microbiology and Human Nutrition, University of Life Sciences in Lublin, Skromna Str. 8, 20-704 Lublin, Poland; piotr.jarocki@up.edu.pl; 3 Department of Food Technology and Human Nutrition, Faculty of Technology and Life Sciences, University of Rzeszow, 4 Zelwerowicza St., 35-601 Rzeszow, Poland; nzurek@ur.edu.pl (N.Ż.); ikapusta@ur.edu.pl (I.K.)

**Keywords:** medicinal mushrooms, triterpenoids, antioxidant activity, anti-inflammatory activity, lipoxygenase, enzyme inhibition, xanthine oxidase, multistep extraction

## Abstract

Oxidative stress and inflammation play a key role in many diseases. This study evaluated the potential of bioactive compounds from Red-belted Bracket and Artist’s Bracket mushrooms to mitigate these processes. Multistep extraction yielded fractions with diversified composition (triterpenoids, polysaccharides) and bioactivities, including antioxidant properties and inhibition of pro-inflammatory enzymes. Both species were rich in triterpenoids: ethanolic extracts from Artist’s Bracket contained mainly ganoderenic and ganoderic acids (≈31 μg/g d.w.), while Red-belted Bracket extracts contained phenolic acids (≈20 μg/g d.w., mainly vanillic and chebulic acids) and triterpenoids (≈73 μg/g d.w., mainly forpinic and formipinic acids). The alkaline and ethanolic extracts exhibited the highest radical scavenging and reducing activities. Lipoxygenase was inhibited only by ethanolic extracts, with IC_50_ values of 0.93 mg d.w./mL for Artist’s Bracket (mixed inhibition) and 0.62 mg d.w./mL for Red-belted Bracket (noncompetitive). Artist’s Bracket was also a potent source of xanthine oxidase inhibitors acting uncompetitively (IC_50_ = 0.71, 1.39, and 2.06 mg d.w./mL for ethanolic, methanolic, and aqueous extracts, respectively). In contrast, Red-belted Bracket was less active (IC_50_ = 3.84 mg d.w./mL, noncompetitive). In conclusion, these mushrooms, particularly their ethanolic extracts, are promising sources of compounds with antioxidant and anti-inflammatory activities, acting as effective inhibitors of lipoxygenase and xanthine oxidase.

## 1. Introduction

Mushrooms and their components play an essential role in dietetics and pharmacology due to their unique chemical composition, which determines a range of health-promoting properties [1,2]. Numerous studies indicate that mushrooms exhibit antioxidant, anti-inflammatory, antidiabetic, and anticancer activities. This activity mainly results from the presence of various groups of bioactive compounds in the fruiting bodies, such as terpenoids, nucleotides, polyphenols, lipid derivatives, and functional polysaccharides. Mushroom polysaccharides, mainly β-glucans, modulate the immune system, supporting the body’s response to infectious and cancerous agents [3]. Terpenoids and polyphenols, in turn, show strong antioxidant properties, neutralising free radicals and protecting cells from oxidative stress. Thanks to this broad spectrum of activity, mushrooms are a valuable component of a functional diet and a potential source of bioactive compounds for the development of new pharmaceutical preparations [4].

Chronic oxidative stress and the associated inflammatory state play a key role in the aetiology of numerous civilisation-related diseases. Inflammation represents a physiological response of the body to tissue damage, infection, or oxidative stress and is mediated by chemical factors, including pro-inflammatory cytokines (e.g., TNF-α, IL-1β, IL-6) and pro-inflammatory enzymes (e.g., LOX, XO). LOX catalyses the conversion of polyunsaturated fatty acids into eicosanoids, which intensify the inflammatory response. At the same time, XO generates reactive oxygen species (ROS) that damage tissues, thereby activating NF-κB and MAPK signalling pathways [5]. Regulating the activity of these enzymes is an essential target of pharmacological anti-inflammatory therapy and also a significant area of interest for modern food technology (functional food development) [6,7]. In light of this, studying enzyme inhibition kinetics is a crucial step toward a deeper understanding of the mechanisms regulating enzyme activity. It also facilitates the optimisation of enzymatic reactions and the design of effective inhibitors to modulate anti-inflammatory effects in biological systems. Although the Lineweaver–Burk method can introduce errors at low substrate concentrations, potentially leading to inaccurate estimates of Km and Vmax, it remains a valuable and simple tool for studying enzyme kinetics. Due to its widespread use, it allows comparison of results with previous studies and, in the case of inhibition, provides valuable insight into the mechanism by which a compound interacts with the enzyme, e.g., by blocking the active site or binding at allosteric/inhibitory sites [8].

Red-belted Bracket (*Fomitopsis pinicola* (Sw.) P. Karst.) and Artist’s Bracket (*Ganoderma applanatum* (Pers.) Pat.) are distinguished from other mushrooms and plants by the presence of specific secondary metabolites with documented biological activity. These include β-glucans (e.g., aplanan, specific β-1,3 and β-1,3/1,6), terpenoids (e.g., ganoderic acids, lanostane-type triterpenoids, forpinic acids), sterols (e.g., ergosta-7,22-dien-3β-ol), and, to a lesser extent, phenolic compounds (e.g., *p*-hydroxybenzoic acid and gallic acid derivatives) [9,10,11,12,13,14,15]. The effects of these metabolites are systemic, and both species have been of interest in ethnopharmacology for many years (it has been suggested that the widely occurring *G. applanatum* may serve as a substitute for the rare and expensive *G. lucidum*). The unique composition of these mushrooms, resulting in part from the presence of characteristic terpenoids, provides a basis for a deeper understanding of their properties, including those that may predispose them to be used for enriching food products.

Nevertheless, despite numerous studies confirming their immunomodulatory, antioxidant, and anti-inflammatory activities, many aspects of these species remain uninvestigated. To date, it has been demonstrated that the ethyl acetate and ethanolic extracts of *F. pinicola* have high antioxidant properties (DPPH, ABTS, nitric oxide and OH tests scavenging activity and inhibition of lipid peroxidation) in vitro, as well as inhibit both acute and chronic inflammation induced with croton oil in a mouse model [16]. In turn, ethanolic extracts (70%) exhibit potent free radical scavenging (DPPH, ABTS, and OH tests) and effectively protect proteins against oxidation [9]. Ganoderma triterpenoids attenuate atherosclerotic plaque formation in high-fat diet–fed rabbits by reducing oxidative stress and inflammation, as evidenced by decreased levels of reactive oxygen species (ROS) and malondialdehyde (MDA), resulting from the downregulation of nuclear transcription factor NF-κB p65 and the scavenger receptor LOX-1 [17]. Methanol extract and hot-water extracts improve the survival rate of RAW 264.7 macrophages and inhibit the nitric oxide (NO)-mediated expression of inducible nitric oxide synthase (iNOS) protein after lipopolysaccharide treatment [18]. The cited studies indicate that these mushrooms appear to be a promising source of metabolites for the prevention and treatment of diseases associated with oxidative stress and inflammation, and that, when incorporated into the diet, they could become an important component of population-level prevention.

In the case of mushrooms, the active compounds belong to various chemical groups, which necessitates the use of complex extraction systems for their isolation. Typically, organic solvents (e.g., ethanol, methanol, ethyl acetate) are employed to isolate terpenoids, sterols, peptides, monosaccharides, and polyphenols. In contrast, polysaccharides are obtained using hot-water extraction (β-glucans) or alkaline solvents (α-glucans) [19,20,21,22]. Of course, the composition of the studied fraction strictly determines the activity, as confirmed by many studies [3,7]. Based on the above, a sequential extraction approach was employed in this study to efficiently recover diverse groups of bioactive components, assess their biological activity, and potentially identify those responsible for specific biological effects. Additionally, the proposed extraction model employs solvents (excluding methanol), enabling the production of extracts directly suitable for food and dietary supplement applications. This represents a novel approach, as mushroom research generally relies on highly specialised solvent systems that prevent direct use in human nutrition without further purification. This study aims to evaluate the antioxidant (antiradical and reducing potentials) and anti-inflammatory (inhibition of pro-inflammatory enzyme activity) properties of components isolated from two medicinal mushrooms belonging to the family Fomitopsidaceae. Additionally, evaluating the obtained extracts and validating them using simple and inexpensive assays can assess their potential application in the production of functional foods. Notably, this is the first study to apply a multistep extraction strategy for this purpose and to determine the kinetic parameters of lipoxygenase and xanthine oxidase inhibition for the resulting complex extracts.

## 2. Materials and Methods

### 2.1. Materials and Chemicals

#### 2.1.1. Chemicals and Materials

All analytical-grade chemicals (if not otherwise stated) were purchased from Merck/Sigma-Aldrich (Poznań, Poland). The mushrooms were collected, manually cleaned, freeze-dried at −42 °C (FreeZone 1 Litre, Labconco, Kansas City, MO, USA), ground at 6 °C (2 × 1 min, MRC SM-450C), and stored in polypropylene boxes at −65 °C. Mushrooms were collected in the Forests of Lasy Janowskie (Lublin Province, Poland) in 2025 and authenticated by Prof. Michał Świeca (Mushroom Classifier Certificate of Competency 1/RZ/2024) from the Department of Food Chemistry and Biochemistry, University of Life Sciences in Lublin, Poland. The samples are deposited at the Department of Food Chemistry and Biochemistry (voucher specimens: M_UPL_2024_18 for Artist’s Bracket (*Ganoderma applanatum* (Pers.) Pat.) and M_UPL_2024_19 for Red-belted Bracket (*Fomitopsis pinicola* (Sw.) P. Karst.)). Additionally, the collected material was determined and identified in the microbiological laboratory of the Department of Biotechnology, Microbiology and Human Nutrition at the University of Life Sciences in Lublin (Supplement S1).

#### 2.1.2. Preparation of Mushroom Extracts

Powdered mushrooms were subjected to multistep extraction. In the 1st step, 10 g of lyophilised powder was extracted with 0.6 L of 70% ethanol (30 °C for 60 min, constant shaking at 60 rpm), centrifuged (Centrifuge MPW-352R, 3997× *g*, 10 min, 20 °C) and stored at −65 °C (E1). Next, the residues were re-extracted with 0.6 L of 50% methanol (30 °C for 60 min, constant shaking at 60 rpm), centrifuged (Centrifuge MPW-352R, 3997× *g*, 10 min, 20 °C) and stored at −65 °C (E2). The resulting residues were re-extracted with 0.6 L water (95 °C for 120 min, constant shaking at 60 rpm), centrifuged (Centrifuge MPW-352R, 3997× *g*, 10 min, 20 °C) and stored at −65 °C (E3). Finally, the residues from the 3rd extraction were re-extracted with 2% NaOH (30 °C for 60 min, constant shaking at 60 rpm), centrifuged (Centrifuge MPW-352R, 3997× *g*, 10 min, 20 °C), and after the pH adjustment to 7.0 (6 M HCl), stored at −65 °C (E4).

### 2.2. Analytical Procedures

#### 2.2.1. Analysis of Bioactive Compounds

##### The Folin–Ciocalteu-Reacting Substances (FC-Reacting Substances)

The content of FC-reactive substances, including potential phenolics, was determined according to the method described by Singleton et al. [23] and expressed as gallic acid equivalents (GAE) in mg per g of dry weight (d.w.).

##### Total Terpenoids and Sterols

The sum of terpenoids and sterols was determined with the vanillin–glacial acetic acid solution and expressed as ursolic acid equivalents (UAE) in mg per g of dry weight (d.w.) [24].

##### Saccharides

Free saccharides and total polysaccharide contents were determined using the phenol–sulfuric acid method and expressed as glucose equivalents (GluE) in mg per g of dry weight (d.w.) [25].

##### Untargeted Metabolomics

Extracts were concentrated using a rotary evaporator (R-215 Rotavapor System, Büchi, Switzerland) at 40 °C. The resulting concentrates were subjected to solid-phase extraction (SPE) using a C18 Sep-Pak cartridge (Waters Associates, Milford, MA, USA) previously conditioned with water. The cartridges were initially rinsed with water to remove sugars. Subsequently, phenolic compounds and triterpenoids were eluted with methanol, evaporated to dryness, and reconstituted in 1 mL of methanol.

Determination of polyphenolics and triterpenoids was carried out using the ultra-performance liquid chromatography (UPLC) Waters ACQUITY system (Waters, Milford, MA, USA) [26]. Individual polyphenolic compounds and triterpenoids were characterised based on retention time, mass-to-charge ratio, fragment ions (Supplement S2), and comparison with commercial standards and literature data [9,10,12,14,27,28,29,30,31,32]. Data processing was performed using Waters MassLynx v.4.1 software (Waters, Milford, MA, USA).

#### 2.2.2. Antioxidant Properties

##### Antiradical Properties (ABTS)

The experiments were performed using the ABTS decolourisation assay [33]. Free radical scavenging activity was expressed as Trolox equivalents (mg per g of dry weight, d.w.).

##### Ferric-Reducing Power (RP)

Reducing power was determined according to Oyaizu [34]. It was expressed as Trolox equivalents (mg/g dry weight, d.w.).

#### 2.2.3. Anti-Inflammatory Properties

##### Ability to Inhibit Xanthine Oxidase Activity (LOXI)

The inhibitory activity against lipoxygenase was determined using linoleic acid as a substrate with the Lipoxygenase Inhibitor Screening Assay Kit (No. 760700, Cayman Chemical, Ann Arbor, MI, USA). For inhibition measurements, the enzyme was preincubated with 10 µL of the tested extract for 10 min before substrate addition. One unit (U) of enzyme activity was defined as the amount causing oxidation of 1 µmol of linoleic acid per minute at pH 7.5 and 30 °C. Inhibitory activity was expressed as IU/g dry weight (d.w.), where one IU corresponds to the amount of inhibitor reducing enzyme activity by 1 U [22].

##### Ability to Inhibit Xanthine Oxidase Activity (XOI)

Xanthine oxidase inhibitory activity was determined using xanthine as the substrate, as previously described [35]. The reaction mixture consisted of 120 µL of 1/15 M sodium phosphate buffer, 20 µL of xanthine oxidase (10 µg/mL; X1875, Sigma-Aldrich, Poland), and 20 µL of the substrate (0.015 mmol). Changes in absorbance were monitored at 234 nm for 3 min. For inhibition assays, the enzyme was preincubated with 20 µL of the tested extract for 10 min before substrate addition. One unit of enzyme activity was defined as the amount converting 1.0 µmol of xanthine to uric acid per minute at pH 7.5 and 25 °C. Inhibitory activity was expressed as IU/g dry weight (d.w.), where one IU corresponds to the amount of inhibitor reducing enzyme activity by 1 U.

##### Determination of IC50 Values and the Mode of Inhibition

To determine the IC_50_ value, the relationship between the degree of inhibition of enzyme activity and the concentration of the extract (ranging from 0 to 1 mg d.w./mL) was established. The IC_50_ values were expressed as mg of mushroom dry weight per 1 mL of the reaction mixture. The inhibition model and kinetic parameters of the process were determined using the inhibitor at the IC_50_ concentration, according to the Lineweaver–Burk method [36].

### 2.3. Statistical Analysis

The data distribution was estimated using Shapiro–Wilk’s test. Statistical analysis of the data was performed using Statistica 10 (StatSoft, Tulsa, OK, USA). Analysis of variance (one-way and two-way) and intergroup differences were analysed using Tukey’s HSD post hoc test at *p* ≤ 0.05.

## 3. Results and Discussion

### 3.1. Effect of Multistep Extraction on the Main Active Constituents and Antioxidant Properties

The mushrooms were subjected to a multistep extraction process to effectively isolate different groups of metabolites potentially exhibiting antioxidant and anti-inflammatory properties (Table 1). In the Artist’s Bracket, the highest content of FC-reactive substances was determined after the first extraction step (54% of the total), while a considerable amount was also detected following the alkaline extraction (23% of the total). Application of the multistep extraction procedure to the Red-belted Bracket yielded approximately 29 mg of FC-reactive substances from 1 g of the lyophilised sample. Notably, all applied conditions efficiently released these compounds from the mushroom matrix—in successive stages, 28%, 20%, 17%, and 35% of the total content were isolated. Both species proved to be excellent sources of triterpenoids and sterols, which were effectively extracted using 70% ethanol; however, the Red-belted Bracket contained nearly three times higher amounts of these compounds than the Artist’s Bracket. Both mushrooms contained comparable contents of simple saccharides (mono- and oligosaccharides extractable with organic solvent). Hot-water and alkali extraction steps release polysaccharides from mushrooms; nevertheless, Artist’s Bracket was a significantly richer source of these compounds. In its case, the third and fourth extraction steps yielded approximately 33% and 65% more of these compounds, respectively, than the Red-belted Bracket.

**Figure 1:**
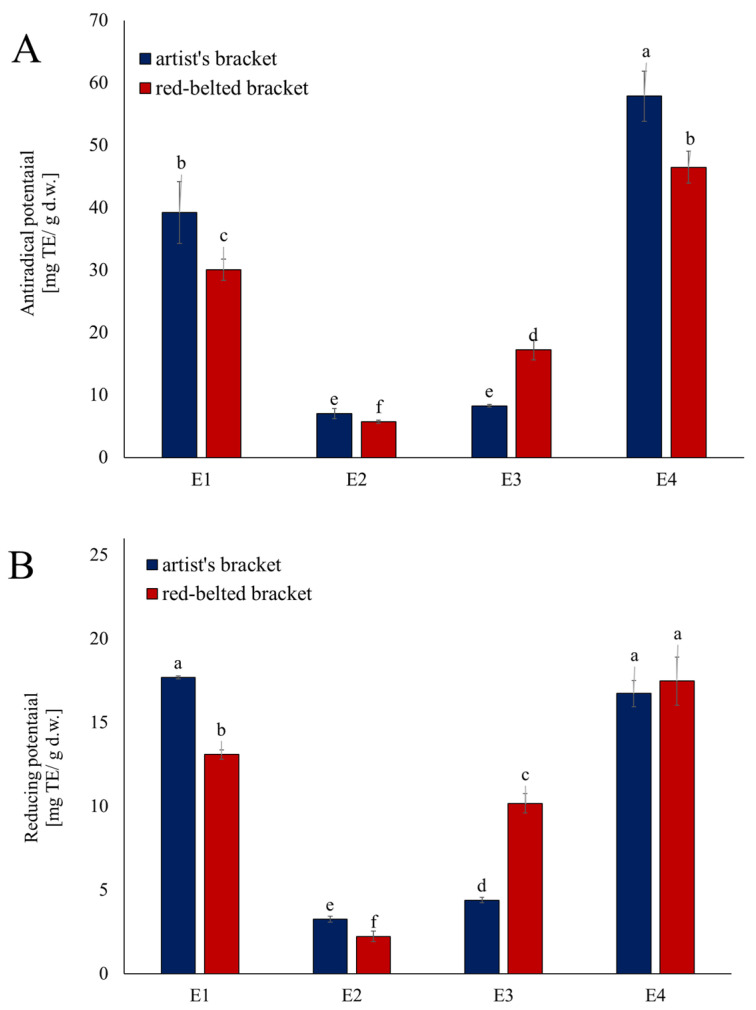
Antiradical (**A**) and reducing (**B**) potential of active fraction from subsequent extraction of mushrooms. Data are mean (*n* = 9) ± SD. Values with the same letters are not significantly different (*p* ≤ 0.05). TE—Trolox equivalents; d.w.—dry weight. E1—70% ethanol extraction, E2—50% methanol extraction, E3—hot-water extraction, E4—NaOH extraction.

The literature data confirm that Polypore exhibits high antioxidant properties; thus, the obtained extracts were evaluated for their antiradical and reducing properties (Figure 1 and Figure S1). The alkaline and ethanolic fractions exhibited the highest antiradical activities. For the Artist’s Bracket, these fractions accounted for 35% and 51% of the total activity (sum of all extracts), respectively. Although the ethanolic fraction from the Red-belted Bracket showed lower activity compared to that of the Artist’s Bracket, the hot-water extract from this mushroom exhibited a considerable ability to quench ABTS radicals (17.2 mg TE/g d.w.) (Figure 1A). A similar trend was observed for reducing power. The alkaline and ethanolic extracts from the Artist’s Bracket exhibited the highest reducing capacity (17.7 and 16.8 mg TE/g d.w., respectively), while the highest value for the Red-belted Bracket was determined in the alkaline extract (17.5 mg TE/g d.w.) (Figure 1B).

Both Artist’s Bracket and Red-belted Bracket are known to be a rich source of active substances, including triterpenoids, phenolics and polysaccharides. In our study, a multistep extraction enabled us to effectively isolate groups of metabolites with distinct chemical characteristics. So far, organic solvents have been used to extract phenolics and terpenoids from mushrooms [37]. The application of methanol for the extraction of lyophilised fruiting bodies of *Ganoderma applanatum* yielded 6.71 mg GAE/g d.w. [13] and 20 mg GAE/g d.w. [38] of FC-reacting substances (potentially phenolics). A high extraction efficiency of terpenoids and phenolics with ethanol as the solvent is also confirmed by the study by Nagadesi and Kannamba [39], who compared this system with methanolic and cold-water maceration. In the aforementioned study, the phenolic content was 85, 58, and 40 mg GAE/g d.w., respectively. Importantly, they proved that both ethanol and 50% methanol used in our study in the 1st and 2nd steps of our extraction process were the most efficient for terpenoids isolation. Considering the Red-belted Bracket, our results are consistent with those reported by Sułkowska-Ziaja et al. [15], who obtained 21.88 mg GAE/g d.w., using a Soxhlet apparatus for extraction. A comparable result was also reported by Onar et al. [40], who found that extraction with 70% ethanol yielded 27.8 mg GAE/g d.w. The use of 70% ethanol in the 1st extraction step in our study yielded 61.5 mg/g d.w. of triterpenes, which is 31% lower than the value reported by Zhang et al. [30]. This variation may be attributed to the use of sonication to enhance extractability rather than to differences in the origin of *F. pinicola* fruiting bodies. 

The use of a sequence of polar solvents in the first and second stages of the multistage extraction likely resulted in the extraction of free sugars and oligosaccharides. This step is commonly applied during polysaccharide extraction as a preliminary purification of the research material to remove lipids, phenols, and triterpenes. Hot-water extraction effectively isolates β-glucans, while alkaline extraction removes polysaccharides poorly soluble in water (glycogen, alkali-soluble β-glucans, chitoglucans) or structural components of the cell wall that form complexes with proteins or lipids. The amount of sugars released with multistep extraction was ca. 5-fold higher (102 vs. 23 mg/g d.w.) than in the study of Iranian *Ganoderma applanatum* [13]. The conditions applied in our study also resulted in a better yield of polysaccharides (70% higher) than in the survey by Kozarsk et al. [41]. Moreover, the obtained extract was characterised by a significantly higher content of Folin–Ciocalteu-reacting substances (potentially polyphenols). Also, in the study conducted by Mohammadifar et al. [13], the total carbohydrate content in the ethanol extract of the Artist’s Bracket (23 mg GluE/g d.w.) was more than two times lower than that determined in our study (60 mg GluE/g d.w.). As shown and discussed, the differences in the determined levels of active compounds are influenced not only by the extraction method but also by environmental conditions and the origin of the research material.

Nevertheless, validating bioactive fractions is essential and plays a crucial role in the conscious application of mushroom extracts in food and medicine, as well as in predicting their health-promoting properties. The relationship between the composition of the fractions (Table 1) and their antioxidant activities (Figure 1) was not strictly linear. It indicates that the observed levels of antioxidant activity cannot be explained solely by the total amounts of individual compounds. Instead, antioxidant capacity appears to be influenced not only by the quantity but also by the qualitative composition of bioactive constituents, including their molecular structures and mutual interactions. As this study was conducted on whole extracts, it is not possible, without additional experiments involving separation and identification of individual compounds, to unequivocally identify which constituents are primarily responsible for the observed activities. However, to further elucidate the relationships, we performed a correlation analysis, which indicated that antiradical activity is mainly associated with saccharides and phenolic compounds (Supplement S3). In the case of Artist’s Bracket fractions, saccharides showed a stronger correlation with antiradical activity (R^2^ = 0.96) than FC-reacting substances (R^2^ = 0.55). Although a detailed qualitative and quantitative analysis of polyphenols and terpenoids (Table 4) confirmed their high content in the ethanolic extract (E1), no compounds from this group were detected in the alkaline extract (E4). These results support the suggestion that the high radical scavenging potential of these extracts is attributable to other groups of compounds, such as β-glucans and proteins. However, despite the strong correlation observed between saccharide content and the antiradical potential of AB, it can be assumed that this relationship is at least partly attributable to the high levels of mono- and disaccharides present in the E1 and E2 extracts, which may have led to an overestimation of the statistical results. In turn, in the E3 and E4 extracts, due to the absence of detectable terpenoids and phenolic compounds, polysaccharides may be responsible for most of the observed effects. It was proven that these conditions allow for the effective isolation of polysaccharides. Also, Ni et al. [42] previously showed that the purified polysaccharides from *Ganoderma applanatum* exhibit dose-dependent antiradical activity (IC50 = 1.5 mg/mL). It seems that in the E1 and E2 extracts, other compounds, e.g., triterpenoids, play a key role. This is also supported by the study of Wang et al. [43], where the isolated monoterpenoids from *G. lucidum* exhibited strong antioxidant properties in the ABTS and ORAC assays.

In contrast, in Red-belted Bracket, antiradical activity was more strongly, positively correlated with FC-reacting substances (R^2^ = 0.91), while saccharides exhibited a moderate correlation (R^2^ = 0.61); terpenoids and sterols appeared to play a marginal role (R^2^ = 0.33). The key role of phenolic acids, which are characterised by high antiradical activity [44], seems to be clear in this case. Regarding ferric-reducing properties, all analysed components in Artist’s Bracket fractions contributed to the observed activity, with strong positive correlations for saccharides (R^2^ = 0.96), FC-reacting substances (R^2^ = 0.81), and terpenoids/sterols (R^2^ = 0.69). In Red-belted Bracket fractions, reducing activity was most strongly correlated with FC-reacting substances (R^2^ = 0.77) and saccharides (R^2^ = 0.78), while a weaker association was observed for terpenoids and sterols (R^2^ = 0.35). These findings further support the conclusion that antioxidant activity in these extracts results from the combined and interacting effects of multiple component groups rather than from a single class of compounds. A detailed analysis of phenolics and terpenoids suggested that the activity of methanolic extracts may be influenced by forpinic acids G and C, which are dominant components of the alcoholic extracts (Table 4). However, without separation and investigation of the activity of purified compounds, this remains speculative. In contrast, the activity of the fraction obtained after hot-water extraction, in addition to the well-documented properties of β-glucans, may be shaped by phenolic acids, including vanillic and chebulic acids.

The antioxidant activity of mushrooms has also been investigated in previous studies using extracts obtained by various methods. Rašeta et al. [45] confirmed the effectiveness of extraction with 70% ethanol and hot water for obtaining *G. applanatum* fractions with high antioxidant potential. In the studies, they found that a high ability to neutralise the DPPH radical was associated with a high content of FC-reacting substances and total sugars. Moreover, the antiradical activity (ABTS assay) of the ethanolic and water extracts was approximately 4-fold and 8-fold higher, respectively, than in our study (160 vs. 35 mg TE/g d.w. and 60 vs. 7 mg TE/g d.w., respectively). Also, the antiradical activity (ABTS test) of *G. applanatum* extracts (ethanolic and hot-water) was significantly higher than in our study (9- and 8-fold, respectively) [46]. On the other hand, the results of reducing power (17.7 mg TE/g d.w.) are nearly the same as those reported by Sułkowska-Ziaja et al. (15 mg TE/g d.w.) [47]. The high reducing potential of the polysaccharide extract from Artist’s Bracket (EC_50_ = 0.18 mg/mL) and Red-belted Bracket observed in our study is consistent with previous observations [41]. Although a direct comparison of the activity of extracts from the studied mushrooms is difficult due to methodological differences (test conditions and extraction procedures), it should be emphasised that our results are consistent with those reported by Li et al. [48] for the ABTS and DPPH assays, where *F. pinicola* was also found to be more effective.

### 3.2. Effect of Multistep Extraction on the Anti-Inflammatory Properties—Inhibition of Lipoxygenase and Xanthine Oxidase

**Figure 2:**
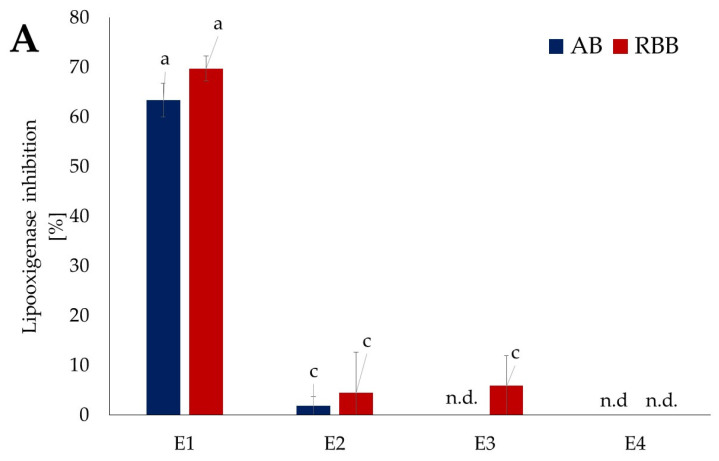

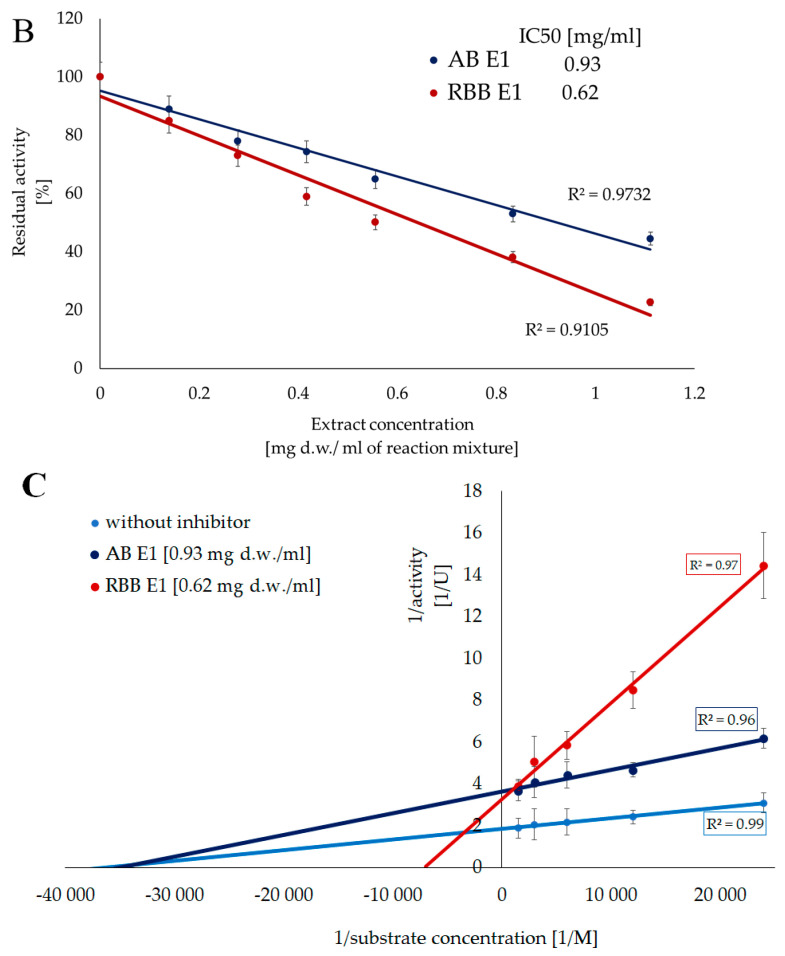
Inhibition of lipoxygenase (LOX) activity by fractions from subsequent extraction of mushrooms. (**A**) LOX inhibition by the fractions from the multistep extraction, (**B**) dose-dependent inhibition (IC50) of LOX by the E1, (**C**) the Lineweaver–Burk plot for the E1 (IC50 concentration). Data are mean (*n* = 9) ± SD. Values with the same letters are not significantly different (*p* ≤ 0.05). d.w.—dry weight; n.d.—lack of activity. AB—Artist’s Bracket; RBB—Red-belted Bracket. E1—70% ethanol extraction, E2—50% methanol extraction, E3—hot-water extraction, E4—NaOH extraction.

Based on the results presented in Table 1, inhibition of lipoxygenase and xanthine oxidase (XO) was observed across different groups of compounds isolated from the studied mushrooms. Lipoxygenases (LOX) play a key role in oxidative stress and inflammatory processes, so in the next step of the study, the obtained extracts were tested in terms of their ability to inhibit lipoxygenase activity (Figure 2). Only the extracts from the 1st step were effective (Figure 2A). Both Artist’s Bracket and Red-belted Bracket had an inhibitory activity at a comparable level (a decrease of ca. 65% of the initial activity of the enzyme); however, a detailed analysis showed that Red-belted Bracket is more effective. The studied extracts showed a dose-dependent effect, allowing determination of IC50 values (Figure 2B). The parameters, 0.93 and 0.62 mg d.w./mL for Artist’s Bracket and Red-belted Bracket, respectively, were used in the further analysis of the inhibition kinetics. According to the Lineweaver–Burk plot, the ethanolic extract from Red-belted Bracket exhibited a mixed type of inhibition (Vmax was decreased, while Km was significantly increased)—the inhibitor can bind to either the free enzyme or the enzyme–substrate complex, which reduces the affinity for the substrate. On the other hand, Artist’s Bracket acted in a noncompetitive mode (Vmax decreased while Km remained unchanged): the inhibitor binds to an inhibitory site on the enzyme or the enzyme–substrate complex without affecting substrate binding. It was reflected in a significant reduction in the turnover number (kcat), which was ca. 50% lower than in the reaction without the inhibitor (Table 2).

**Figure 3:**
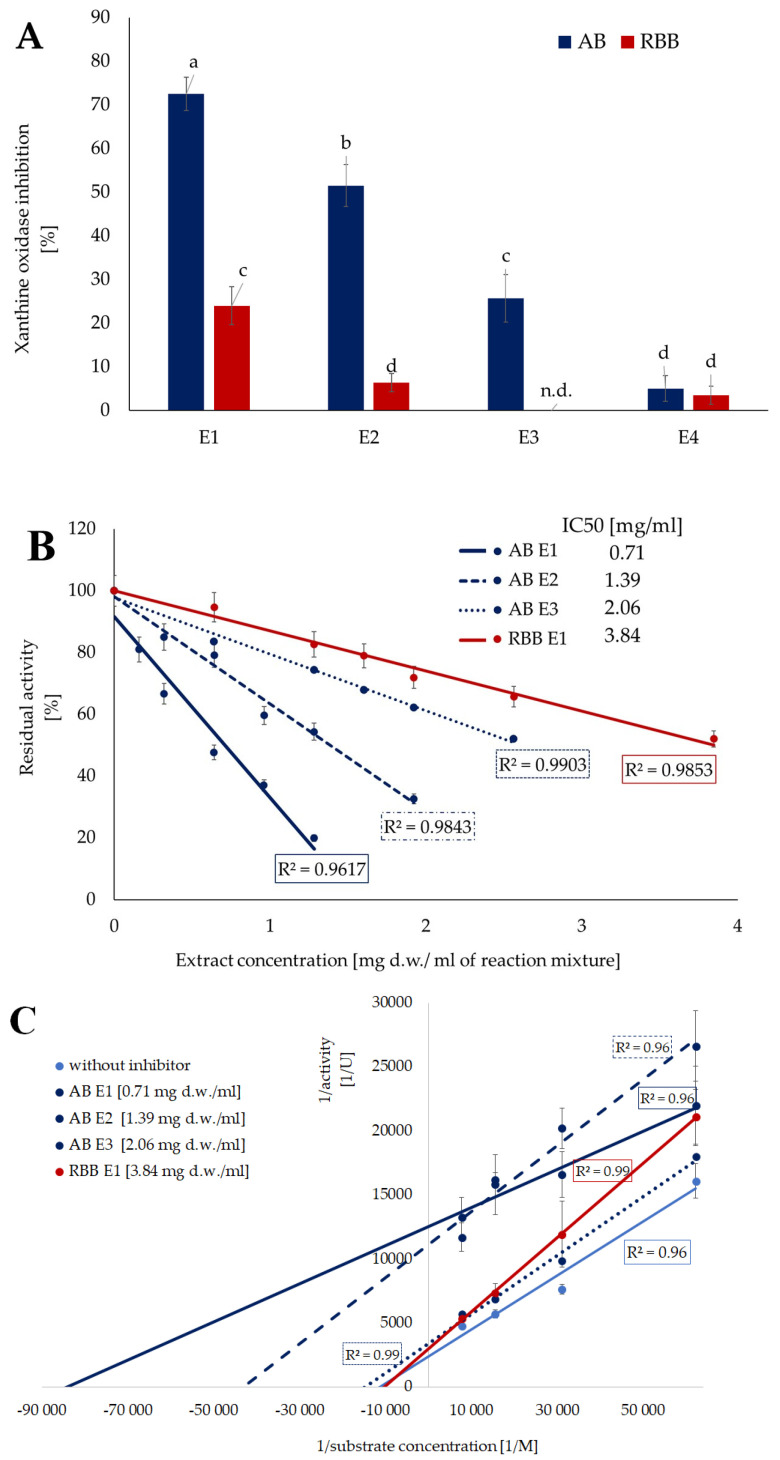
Inhibition of xanthine oxidase (XO) activity by fractions from subsequent extraction of mushrooms. (**A**) XO inhibition by the fractions from the multistep extraction, (**B**) dose-dependent inhibition (IC50) of XO, (**C**) the Lineweaver–Burk plot for the XO inhibition (IC50 concentration). Data are mean (*n* = 9) ± SD. Values with the same letters are not significantly different (*p* ≤ 0.05). d.w.—dry weight; n.d.—lack of activity. AB—Artist’s Bracket; RBB—Red-belted Bracket. E1—70% ethanol extraction, E2—50% methanol extraction, E3—hot-water extraction, E4—NaOH extraction.

In turn, xanthine oxidase, which is one of the primary sources of reactive oxygen species (ROS) in the body, was inhibited by both the ethanol extracts from the two mushrooms and by compounds from the methanolic extracts and the polysaccharide-rich fraction of Artist’s Bracket (Figure 3A). Artist’s Bracket turned out to be an excellent source of compounds inhibiting the activity of this enzyme, with IC_50_ values of 0.71, 1.39 and 2.06 mg d.w./mL for the extracts obtained during the first three stages of isolation, respectively (Figure 3B). Compounds present in this extract act in an uncompetitive mode of action (Vmax and Km were decreased): the inhibitor binds only to the enzyme–substrate complex, which is reflected in decreased substrate affinity. A marked reduction in kcat values indicated an inhibitory effect; in the presence of the ethanolic and methanolic fractions, kcat was approximately 5-fold lower than in the reaction without the inhibitor. Red-belted Bracket was less prominent, and only ethanolic extracts exhibited the inhibitory activity (IC50 3.84 mg d.w./mL) against xanthine oxidase. The compounds present in this extract acted in a noncompetitive manner, without influencing the enzyme’s affinity for the substrate (Km constant) (Figure 3C, Table 3).

It should be emphasised that the study demonstrates only the ability of the tested fractions to modulate markers of inflammation in vitro. The determined IC_50_ values may suggest that these concentrations could be achievable in the gastrointestinal tract, where the compounds may exert local effects, e.g., alleviating inflammatory conditions. Nevertheless, additional in vivo studies are required to provide deeper insight into their health-promoting potential under physiological conditions, where biological activity is usually tailored by dilution of gastrointestinal contents, as well as by bioavailability, bioaccessibility, and metabolism of bioactive compounds [49,50,51].

In the Red-belted Bracket, LOX and XO inhibitory activity showed a very strong positive correlation with the content of terpenoids/sterols (R^2^ = 0.97 and 0.97, respectively) and saccharides (R^2^ = 0.85 and 0.78, respectively) (Supplement S3). However, to date, no inhibition of LOX and XO activities by simple sugars has been reported in the literature. Since these compounds, due to their physicochemical properties, were overwhelmingly present in the first extracted fraction (E1) exhibiting the highest LOX and XO inhibitory activities, it can be assumed that the remaining groups of compounds play a key role in the observed activities. In contrast, in the Artist’s Bracket, the strongest correlations for LOX and XO inhibition were observed for terpenoids/sterols (R^2^ = 0.99 and 0.71) and Folin–Ciocalteu-reacting substances (R^2^ = 0.96 and 0.57), which seems to agree with the literature data presented below.

Previous studies have confirmed the effectiveness of various mushrooms from the order Polyporales in modulating the activity of pro- and antioxidant enzymes. LOX activity was inhibited exclusively by methanolic extracts (10 mg/mL) of *Trametes hirsuta*, *Trametes orientalis*, *Daedaleopsis confragosa*, and *Roseofomes subflexibilis*, which reduced its activity by 31.9%, 33.4%, 42.2%, and 43.4%, respectively. In the aforementioned study, *F. pinicola* and *F. nigra* had activities of 3% and 43%, respectively [52]. They also showed that inoscavin A, isolated from *Phellinus baumii*, exhibits a strong inhibitory activity against LOX, with an EC_50_ of 6.8 μM. Also, in the study by Nong et al. [53], it has been demonstrated that the IC_50_ values of ganoderic acids A, B, C2, D2, and F were 16.5, 6.9, 8.3, 9.3, and 14.3 μg/mL, respectively.

Similarly, studies on XO activity inhibition, like those on LOX inhibition, remain scarce. In this area, particular emphasis should be placed on a study describing the activity of 47 native, wild Hungarian mushrooms [54]. The mushrooms with the most vigorous XO inhibitory activity included *Hypholoma fasciculare*, *Suillus grevillei*, and *Tricholoma populinum*. The methanolic extract of *Hypholoma fasciculare* exhibited significant XO inhibition (77.7%, IC_50_ = 67.8 μg/mL). Reported IC50 values were significantly lower than those determined in our study; however, it should be emphasised that in the cited studies, these values referred to the dried extract, whereas in the present study, they refer to the dry weight of the mushroom from which the extract was obtained. The XO inhibitory activity of some species, *Fistulina hepatica*, *Lentinus lepideus*, *Phellinus linteus*, and *Pleurotus cornucopiae*, among others, has been confirmed [41,55,56,57]; unfortunately, the results cannot be directly compared due to differences in the methods (extract preparation and expression of activity). Nevertheless, the common denominator across the different extraction methods is the high activity of the fractions obtained with methanol or ethanol. Therefore, we attempted to determine the quantitative and qualitative composition of the extracts used in this study (Table 4).

Ethanolic extracts (E1) of Red-belted Bracket contained two main groups of active compounds: phenolics and terpenoids. Among phenolics, vanillic acid and chebulic acids (benzopyran tannin) were dominant. Although phenolic acids were not present in the methanolic extract (E2), hot-water extraction (E3) enabled their release from the matrix. The determined profile of phenolic acids is similar to that recorded previously by Sułkowska-Ziaja et al. [47]; however, the amounts are significantly lower, which may result from the applied extraction conditions—in the cited study, the material was subjected to acid hydrolysis, enabling the isolation of bound phenolic acids. The presence of phenolic acids, including protocatechuic acid, p-hydroxybenzoic acid, and gallic acid, in this species was also confirmed [58]. The cited study also reported significant levels of gallic and ellagic acids (not detected in our samples); however, this may be due to the detection conditions used, as chebulic acids and gallic acids exhibit similar ion-fragmentation patterns. In the extracts from the first two steps, a significant amount of terpenoids was also detected, with forpinic acids dominant, which is in line with the previous studies [9,12,30]. In the studied extracts of Artist’s Bracket (except E4), only ganoderenic acids were detected, which was consistent with earlier studies on fingerprinting and the quality evaluation of Ganoderma Spp. [14]. Their level was highest in the ethanolic extract (E1), where it accounted for over 88% of the total content, whereas in the methanolic (E2) and aqueous (E3) extracts, their proportion was significantly lower, amounting to 8.2% and 3.4% of the total, respectively. We did not detect phenolic compounds, which are commonly reported for this genus (also in our study), using the unspecific FC-test [41,58]. This group of functional metabolites has also been identified using more sophisticated techniques (e.g., LC-MS/MS); however, their levels were marginal [58] and seem to be directly related to compounds absorbed from wood rather than synthesised de novo.

In our study, IC_50_ values were expressed as mg of mushroom dry weight per mL because, for complex extracts (not purified compounds), these units seem more appropriate and practical for comparing extract activities and potential food-related applications. Simultaneously, it should be noted that these values cannot be directly comparable to IC_50_ data reported in μM or μg/mL without conversion and normalisation, given the extraction methodology. Nevertheless, in our opinion, this approach to presenting the results allows for comparison and positions our mushrooms (extracts) in the context of other studies.

In our studies, it should be noted that in complex extracts, the observed activity results from the combined effects of all compounds; therefore, it is not possible to identify the key compounds responsible for the activity unequivocally. Nevertheless, the detailed identification of the components of the most active fraction from the studied mushrooms allows for a deeper discussion of the pro-oxidative enzyme-inhibition process. Previous studies have shown that the activity of lipoxidase and xanthine oxidase may be effectively inhibited by both phenolics and terpenoids. In the study by Łyko et al. [59], dried terpenoid-rich extracts of *Rhododendron luteum* obtained after supercritical fluid CO_2_ (30% ethanol) extraction inhibited LOX and XOI in a competitive mode of action, with IC50 values of 0.5 and 2.36 mg/mL, respectively. This activity is slightly higher than that determined in our study for the ethanolic extract; however, it should be noted that in this study, IC50 values were expressed per gram of dry mushroom used for extraction. Also, ferulic, syringic and trans-cinnamic acids [60] and rutin [61] acted as inhibitors of LOX, with an uncompetitive mode of action observed for the phenolic acids. Huang et al. [62] showed that terpenoids from *Pistacia chinensis* leaf inhibit xanthine oxidase according to a mixed type of inhibition. So far, reports on the modulation of enzyme activity by mushroom components are scarce, especially in terms of describing the kinetics of the process. Still, they generally confirm that terpenoid fractions are the most active. Previously, sesquiterpenoids from *Fomitopsis pinicola* exhibited the most significant inhibition of superoxide anion generation and elastase release with IC50 values of 0.81 and 0.74 mM, respectively [63].

The ethyl acetate extracts of *Cordyceps militaris* possessed the highest XO inhibitory properties, and it was suggested that the activity is responsible for lipid derivatives (pentadecanal, hexadecanoic acid, methyl 2-oxohexadecanoate and N-(2-Hydroxyethyl) octanamide) and 3′-deoxyadenosine (cordycepin)—after fractionation, the lowest IC50 (68 μg of dried extract/mL) was determined for the 10th fraction containing tetradecanal, pentadecanal and 3′-deoxyadenosine. Amagata et al. [64] suggested that sponge-derived terpenoids, being LOX inhibitors, acted with redox (operate by reducing lipoxygenase to its inactive, ferrous form) and non-redox (enzyme inactivation occurs by competitive or allosteric inhibition) mechanisms. It may be suggested that the second mechanism is involved in the ethanolic extracts of *F. pinicola* and *G. applanatum*; however, an unambiguous statement is difficult to make because the samples examined are mixtures of multiple compounds. A similar situation is observed in our study for XO inhibition, where uncompetitive inhibition has been identified. In turn, tsugaric acid (lanostanoids) isolated from the fruit bodies of *Ganoderma tsugae* exhibited significant inhibitory effects on xanthine oxidase (XO) activity with an IC50 value ranging from 90.2 to 182 μM [65]. The purified tsugaric acid D (3α-acetoxy-22-oxo-5α-lanosta-8,24-dien-21-oic acid) inhibited XOI in a dose-dependent manner according to a competitive mode (Ki 0.6 μM). They suggested that, in addition to the chemical skeleton and conformation of the compound, a carbonyl group, including carbonyl in carboxylic acid, acyl group or ester group, may interfere with the interaction between the enzyme and the enzyme–substrate complex. Due to a structural similarity of tsugaric acid D to forpinic acids from Red-belted Bracket and ganodermic acids from Artist’s Bracket, an analogical mechanism of inhibitory action may be postulated. It is also confirmed by the screening study of potential XO inhibitors from *G. leucocontextum* using the affinity ultrafiltration method [66].

Triterpenoids, including ganoderic acid A, ganoderic acid D, ganodermanontriol, and ganoderal A, demonstrated significant affinity for native XO; the mechanism was described by molecular docking analysis. They showed that among the studied compounds, ganodermic acids had the highest affinity for the enzyme (intermolecular energy approximately −9.5 kcal/mol) and interact with the enzyme via hydrophobic interactions, hydrogen bonding, and salt bridges. Ganodermic acid A interacts with a region of the enzyme that does not directly participate in catalysis (I264, R394, K395). In contrast, ganodermic acid D appears to modify the catalytic activity of the molybdenum domain (Mo-cofactor domain) and to interact with amino acids that stabilise the structure and charge of the active site (W336, F337, A338, I358, and D360). The Lineweaver–Burk double-reciprocal plot showed that, as in our studies, ganodermic acid acts as a noncompetitive inhibitor with a Ki of 84 µg/mL (~163 µM).

## 4. Conclusions

The results confirmed that Red-belted Bracket and Artist’s Bracket possess significant antioxidant and anti-inflammatory properties, which may support their use in anti-gout therapy. Significantly, the activity is strongly determined by the extraction method used, as different groups of active compounds (terpenoids, polysaccharides) require distinct processing conditions. We also demonstrated that commonly used spectrophotometric methods, although useful for raw material validation, provide incomplete information regarding the composition of active fractions.

As shown, the highest activity was observed in the ethanolic fraction, which, in the case of Red-belted Bracket, contained both polyphenols and terpenoids, whereas in Artist’s Bracket, it contained only terpenoids. The study also provides valuable insights into the kinetics of pro-oxidative enzyme inhibition. The mixture of Artist’s Bracket terpenoids acts competitively with LOX, whereas XO is inhibited uncompetitively. In contrast, the ethanolic extract of Red-belted Bracket inhibited LOX via a mixed-type mechanism, while XO was inhibited through a noncompetitive mechanism. Despite the significance of the obtained results, particularly regarding the inhibition of pro-oxidant enzymes, certain limitations should be noted. In particular, further studies are required to elucidate the relationships between the activity of individual compounds present in the extracts, including fractionation and interaction analyses (e.g., isobolography), as well as the variability in the quantitative and qualitative composition of the extracts associated with the different origins of the studied mushrooms. The novelty of this study lies in the simultaneous comparison of two mushroom species in terms of their antioxidant and anti-inflammatory properties. We not only evaluated the biological activity of the extracts but also precisely identified and quantified the bioactive compounds present, while attempting to correlate specific activities with key constituents. This research represents a valuable attempt to validate these raw materials for potential use in the production of functional foods and dietary supplements, thereby linking detailed chemical characterisation with practical application potential. Additionally, information on the activity of the whole mixture, combined with the low cost of obtaining the extracts, opens new possibilities for the application of these mushrooms in the prevention of lifestyle-related diseases.

## Figures and Tables

**Table 1:** Content of main bioactive components in the fractions from subsequent extractions of mushrooms.

		Artist’s Bracket	Red-Belted Bracket
FC-reacting substances [mg GAE/g d.w.]	E1	10.1 ± 0.12 a	8.11 ± 0.17 b
	E2	1.82 ± 0.04 g	5.96 ± 0.19 c
	E3	2.45 ± 0.09 f	5.07 ± 0.05 d
	E4	4.28 ± 0.11 e	10.15 ± 0.11 a
	Sum	18.63	29.29
Total terpenoids and sterols [mg UAE/g d.w.]	E1	29.9 ± 1.23 b	61.5 ± 2.61 a
	E2	2.22 ± 0.22 e	6.75 ± 0.39 d
	E3	2.58 ± 0.48 e	4.98 ± 0.83 d
	E4	5.09 ± 0.26 d	14.41 ± 0.34 c
	Sum	39.86	87.64
Mono- and oligosaccharides [mg GluE/g d.w.]	E1	53.3 ± 2.08 b	50.4 ± 3.03 b
	E2	7.15 ± 1.33 e	4.75 ± 0.95 e
	Sum	60.4	55.2
Polysaccharides [mg GluE/g d.w.]	E3	28.7 ± 0.92 c	19.2 ± 1.86 d
	E4	77.7 ± 0.68 a	27.2 ± 2.03 c
	Sum	106.4	46.4

Data are mean (*n* = 9) ± SD. Values for the features with the same letters are not significantly different (*p* ≤ 0.05). UAE—ursolic acid equivalents; GAE—gallic acid equivalents; GluE—glucose equivalents; d.w.—dry weight. E1—70% ethanol extraction, E2—50% methanol extraction, E3—hot-water extraction, E4—NaOH extraction.

**Table 2:** Kinetic parameters of lipoxygenase inhibition by ethanolic extracts (E1) from the mushrooms.

	Without Inhibitor	Artist’s Bracket	Red-Belted Bracket
Vmax[mU]	536 ± 14.1 a	274 ± 16.6 b	272 ± 17.7 b
Km[μM]	26.8 ± 0.70 b	231.7 ± 1.66 a	27.4 ± 2.94 b
Mode of inhibition	-	noncompetitive	mixed
k_cat_ [s^−1^]	8.40 × 10^8^ a	4.29 × 10^8^ b	4.26 × 10^8^ b

Values with the same letters are not significantly different (*p* ≤ 0.05). Vmax—maximum reaction velocity, Km—Michaelis constant, k_cat_—turnover number (catalytic constant).

**Table 3:** Kinetic parameters of xanthine oxidase inhibition by extracts from the mushrooms.

	Without Inhibitor	Artist’s Bracket			Red-Belted Bracket
		E1	E2	E3	E1
Vmax[U]	0.415 ± 0.023 a	0.080 ± 0.010 d	0.090 ± 0.010 d	0.292 ± 0.017 c	0.335 ± 0.026 b
Km[μM]	87.7 ± 4.8	11.9 ± 1.5	23.1 ± 0.0	67.0 ± 0.0	97.2 ± 7.6
Mode of inhibition	-	uncompetitive	uncompetitive	uncompetitive	noncompetitive
k_cat_ [s^−1^]	0.072	0.014	0.016	0.051	0.058

Values with the same letters are not significantly different (*p* ≤ 0.05). E1—70% ethanol extraction, E2—50% methanol extraction, E3—hot-water extraction. Vmax—maximum reaction velocity, Km—Michaelis constant, k_cat_—turnover number (catalytic constant), k_cat_/Km—catalytic efficiency.

**Table 4:** Individual phenolics and terpenoids in the extracts of the Artist’s Bracket and Red-belted Bracket.

	Compound [μg/g d.w.]	E1	E2	E3	E4
	Red-belted Bracket				
1.	Vanillic acid	5.02 ± 0.054 a	Tr.	3.60 ± 0.12 b	Tr.
2.	Protocatechuic acid	2.26 ± 0.068	Tr.	Tr.	0.00
3.	Sinapic acid	1.81 ± 0.042 b	Tr.	2.76 ± 0.103 a	0.00
4.	Quercetin 3-*O*-rutinoside	1.40 ± 0.013 a	Tr.	1.09 ± 0.045 b	0.00
5.	Rosmarinic acid glucoside	1.23 ± 0.013 b	Tr.	2.07 ± 0.081 a	0.00
6	Ferulic acid	3.39 ± 0.074	Tr.	Tr.	0.00
7.	Chebulic acid	5.92 ± 0.104 a	Tr.	2.24 ± 0.19 b	Tr.
8.	6-α-hydroxy-3.16-dioxolanosta-7(8).9(11).24-trien-21-oic acid	3.43 ± 0.040	Tr.	Tr.	0.00
9.	Dehydrotumulosic acid	1.36 ± 0.045	Tr.	Tr.	0.00
10.	16-α-hydroxy-3. oxolanosta-7.9(11).24-trien-21-oic acid	2.20 ± 0.004	Tr.	Tr.	0.00
11.	Irpeksolactin E	2.90 ± 0.012	Tr.	Tr.	0.00
12.	Forpinic acid D	1.98 ± 0.014	Tr.	Tr.	0.00
13.	Forpinic acid E	1.15 ± 0.014	Tr.	Tr.	0.00
14.	16-α-hydroxy-dehydrotraumetenolic acid	2.71 ± 0.017	Tr.	Tr.	0.00
15.	Unspecified	5.27 ± 0.032	Tr.	Tr.	0.00
16.	Formipiniate B	2.48 ± 0.037	Tr.	Tr.	0.00
17.	Forpinic acid A	2.44 ± 0.057	Tr.	Tr.	0.00
18.	20-OH-lucidenic acid A	1.56 ± 0.039	Tr.	Tr.	0.00
19.	Forpinic acid F	1.90 ± 0.007	Tr.	Tr.	0.00
20.	Forpinic acid G	20.9 ± 0.18 a	5.20 ± 0.14 b	Tr.	0.00
21.	Forpinic acid C	12.0 ± 0.38 a	3.76 ± 0.098 b	Tr.	0.00
22.	Piptolinic acid D	1.97 ± 0.025	Tr.	Tr.	0.00
23.	Formipinic acid H	3.52 ± 0.038	Tr.	Tr.	0.00
24.	Unspecified	2.27 ± 0.068	Tr.	Tr.	0.00
25.	Formitopsic acid F	2.69 ± 0.062	Tr.	Tr.	0.00
	Artist’s Bracket				
1.	Ganoderenic acid A	7.78 ± 0.085 a	1.56 ± 0.012 b	0.73 ± 0.082 c	Tr.
2.	Ganoderenic acid D	2.10 ± 0.063 a	0.40 ± 0.091 b	0.19 ± 0.011 c	0.00
3.	Ganoderic acid C6	3.57 ± 0.082 a	Tr.	Tr.	0.00
4.	Ganoderic acid A	3.31 ± 0.031 a	0.66 ± 0.021 b	0.18 ± 0.021 c	0.00
5.	Ganoderic acid H	3.15 ± 0.033 a	Tr.	Tr.	0.00
6.	Ganoderic acid F	8.33 ± 0.183 a	Tr.	Tr.	0.00

Data are mean (*n* = 9) ± SD. Values with the same letters are not significantly different (*p* ≤ 0.05). d.w.—dry weight. Tr.—trace. E1—70% ethanol extraction, E2—50% methanol extraction, E3—hot-water extraction, E4—NaOH extraction.

## Data Availability

The data that support the findings are available in RepOD: https://doi.org/10.18150/SYQWDX.

## Supplementary Materials

The following supporting information can be downloaded at: https://www.mdpi.com/article/10.3390/antiox15060663/s1, Supplement S1. Genetic identification of mushrooms. Figure S1. Radical scavenging activity kinetics. Supplement S2. Identification parameters for UPLC–MS analysis of phenolics and terpenoids. Supplement S3. Relationship between the content of active compounds and bioactivity (correlation coefficient).
