# Supplementary material for: Lipoxygenase and Xanthine Oxidase Inhibition and Antioxidant Potential of Fractions Obtained by Multistep Extraction of Artist’s Bracket (Ganoderma applanatum (Pers.) Pat.) and Red-Belted Bracket (Fomitopsis pinicola (Sw.) P. Karst.)
Source: Antioxidants (Basel). 2026 May 25;15(6):663. doi: 10.3390/antiox15060663 (PMC13295654; doi:10.3390/antiox15060663)
Supplement: Supplementary file 1 [file antioxidants-15-00663-s001.zip › Figure S1. Radical scavenging activity-kinetics.pdf]

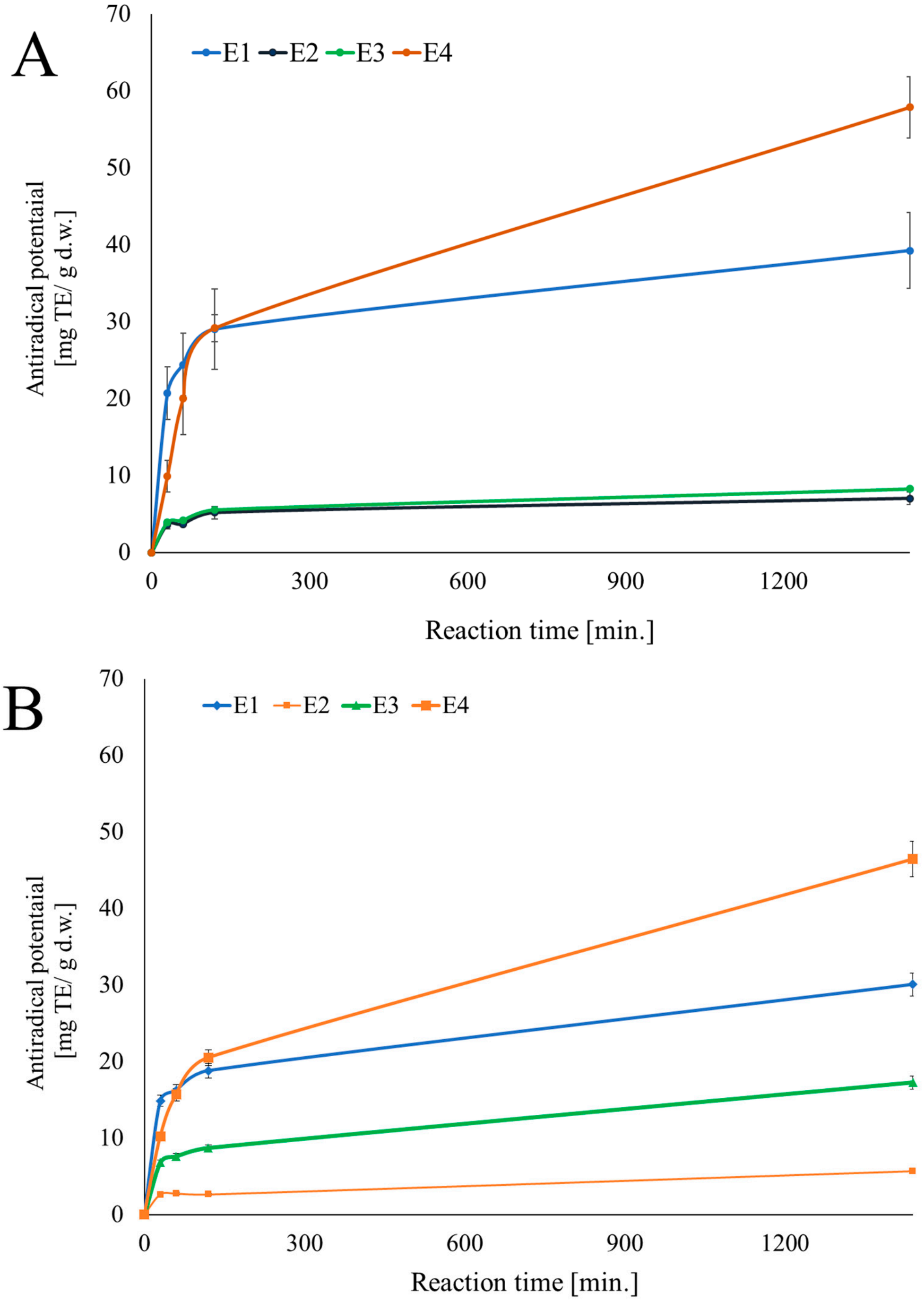

Figure S1. Kinetics of ABTS radicals scavenging

A- Artist's bracket; B- Red-belted bracket; TE- Trolox equivalents; d.w.- dry mass. E1- 70% ethanol extraction, E2- 50% methanol extraction, E3- hot-water extraction, E4- NaOH extraction.
