# Supplementary material for: Lipoxygenase and Xanthine Oxidase Inhibition and Antioxidant Potential of Fractions Obtained by Multistep Extraction of Artist’s Bracket (Ganoderma applanatum (Pers.) Pat.) and Red-Belted Bracket (Fomitopsis pinicola (Sw.) P. Karst.)
Source: Antioxidants (Basel). 2026 May 25;15(6):663. doi: 10.3390/antiox15060663 (PMC13295654; doi:10.3390/antiox15060663)
Supplement: Supplementary file 1 [file antioxidants-15-00663-s001.zip › Suppl. S1. Genetic identification of mushrooms.pdf]

Materials and methods

Species identification of *Fomitopsis* sp. M_UPL_2024_19 and *Ganoderma* sp. M_UPL_2024_18 was performed based on the analysis of the rRNA internal transcribed spacer (ITS) region. Genomic DNA was extracted using the Plant & Fungi DNA Purification Kit (Eurx) following the manufacturer's instructions, and the concentration and purity of the nucleic acids were assessed using a NanoDrop 2000c spectrophotometer (Thermo Scientific). The amplification of the ITS region was carried out in a T100 thermocycler (Bio-Rad) using 2× DreamTaq PCR Master Mix (Thermo Scientific), 1 μM of each primer (ITS1: 5′-TCCGTAGGTGAACCTGCGG-3′ and ITS4: 5′-TCCTCCGCTTATTGATGC-3′), and 50 ng of template DNA in a final volume of 20 μl. The PCR cycling profile consisted of an initial denaturation at 95 °C for 3 minutes, followed by 40 cycles comprising denaturation at 95 °C for 30 seconds, primer annealing at 60 °C for 30 seconds, and extension at 72 °C for 30 seconds, with a final elongation step at 72 °C for 8 minutes. The obtained PCR products were verified by agarose gel electrophoresis and subsequently analysed using a 3730xl DNA Analyzer (Life Technologies). The resulting ITS sequences were compared against the NCBI GenBank database using the BLAST algorithm, and a phylogenetic tree was constructed using MEGA 11 software.

Results

The obtained sequences were analyzed against the NCBI sequence databases. For *Fomitopsis* sp. M_UPL_2024_19, the closest related isolates belonged to the species *Fomitopsis pinicola* and *Fomitopsis subpinicola* (sequence similarity of 99.85%). Slightly lower similarity levels were observed for *Fomitopsis hengduanensis* (99.53%), *Fomitopsis abieticola* (99.06%), and *Fomitopsis massoniana* (98.90%). Phylogenetic analysis based on the selected ITS sequences from the NCBI databases and the sequences obtained in this study is presented in Figure S1A. In the case of *Ganoderma* sp. M_UPL_2024_18, identical sequences were found in several isolates of *Ganoderma applanatum*. A highly similar sequence was also observed for *Ganoderma alpinum* BJFC 034326 (99.64% sequence similarity), although this alignment showed a lower sequence coverage of 86%. Phylogenetic analysis based on the selected ITS sequences from the NCBI databases and the sequences obtained in this study is presented in Figure S1B.

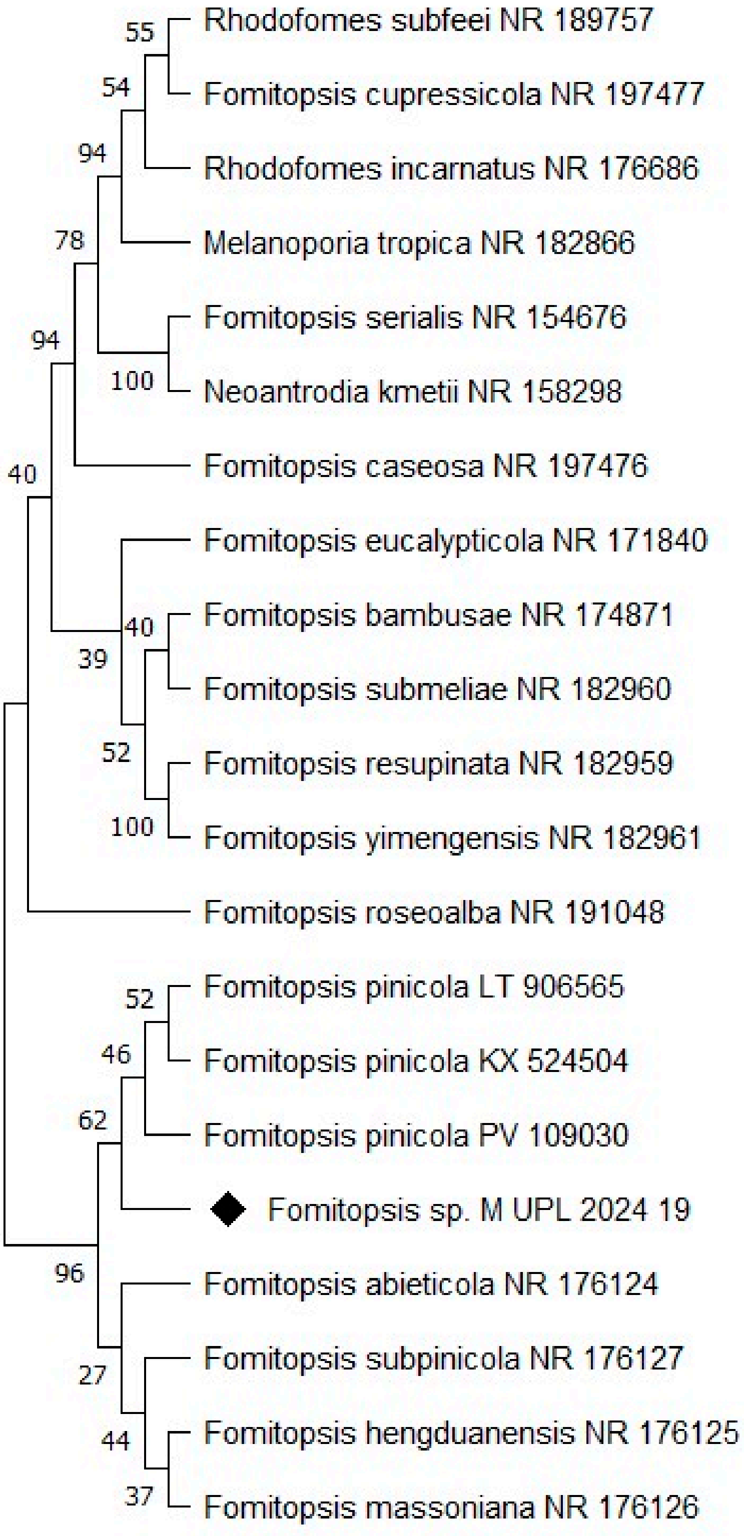

Figure S1A

Phylogenetic tree based on rRNA ITS region prepared for *Fomitopsis* sp. M_UPL_2024_19 and selected members belong to *Fomitopsidaceae*. The tree was constructed using the neighbour-joining method with the software package MEGA version 11.0.

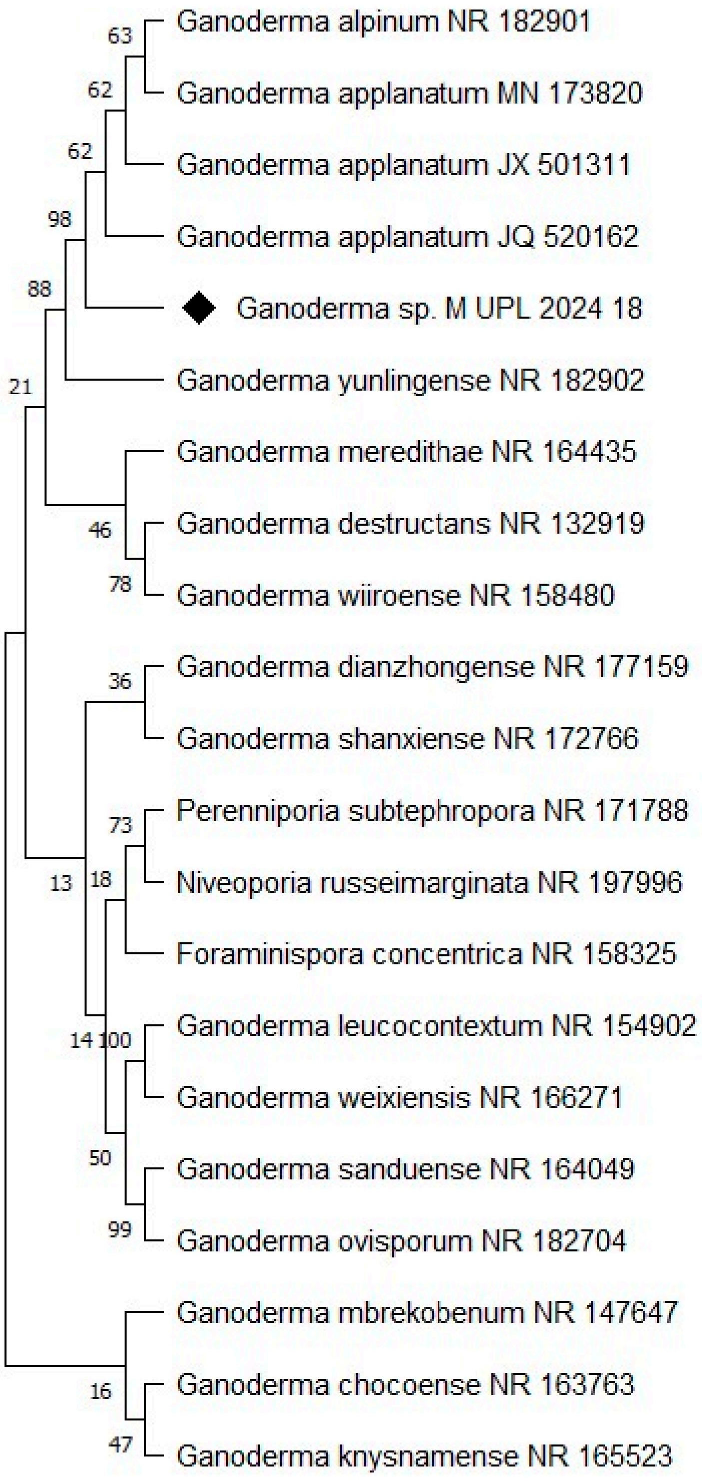

Figure S1B

Phylogenetic tree based on the rRNA ITS region showing the position of *Ganoderma* sp. M_UPL_2024_18 among selected isolates belonging to the family *Ganodermataceae*. The tree was constructed using the neighbour-joining method implemented in the MEGA version 11.0 software package.
