# Supplementary material for: Lipoxygenase and Xanthine Oxidase Inhibition and Antioxidant Potential of Fractions Obtained by Multistep Extraction of Artist’s Bracket (Ganoderma applanatum (Pers.) Pat.) and Red-Belted Bracket (Fomitopsis pinicola (Sw.) P. Karst.)
Source: Antioxidants (Basel). 2026 May 25;15(6):663. doi: 10.3390/antiox15060663 (PMC13295654; doi:10.3390/antiox15060663)
Supplement: Supplementary file 1 [file antioxidants-15-00663-s001.zip › Suppl. S2. Identification parameters for UPLC–MS analysis of phenolics and terpenoids.pdf]

Suppl. S2. Identification parameters for UPLC–MS analysis of phenolics and terpenoids.

Red-belted Bracket					
Compound		Rt	λ_{max}	[M-H] m/z	
		min	nm	MS	MS/MS
Phenolics					
1.	Vanillic acid	1.67	274	167^-	123
2.	Protocatechuic acid	1.76	259, 300	153^-	109
3.	Sinapic acid	1.92	324	223^-	164
4.	Quercetin 3-*O*-rutinoside	2.45	255, 355	609^-	301
5.	Rosmarinic acid glucoside	2.70	324	521^-	359
6	Ferulic acid	2.87	327	193^-	134
7.	Chebulic acid	2.93	320, 402	355^-	337, 261
Terpenes					
8.	6-α-hydroxy-3,16-dioxolanosta-7(8),9(11), ,24-trien-21-oic acid	6.43	-	481^-	463, 437, 403, 388, 373
9.	Dehydrotumulosic acid	6.55	-	483^-	465, 421, 255
10.	16-α-hydroxy-3, oxolanosta-7,9(11),24- -trien-21-oic acid	6.60	-	467^-	423, 407, 389, 373, 311
11.	Irpeksolactin E	6.64	-	485^-	467, 423, 407, 353, 337
12.	Forpinic acid D	6.85	-	479^-	465, 435, 441
13.	Forpinic acid E	6.98	-	495^-	465, 421, 405, 391, 373
14.	16-α-hydroxy-dehydrotraumetenolic acid	7.06	-	469^-	467, 425, 409, 391, 337
15.	Unspecified	7.38	-	467^-	-
16.	Formipiniate B	7.43	-	525^-	497, 483, 465, 441
17.	Forpinic acid A	8.52	-	599^-	537, 455
18.	20-OH-lucidenic acid A	8.60	-	455^-	149
19.	Forpinic acid F	8.68	-	639^-	537, 451, 371, 339
20.	Forpinic acid G	8.74	-	583^-	465, 449, 434, 389
21.	Forpinic acid C	8.79	-	541^-	451, 371, 339
22.	Piptolinic acid D	8.87	-	465^-	435, 421, 405, 369
23.	Formipinic acid H	9.00	-	627^-	537, 465, 373
24.	Unspecified	9.10	-	495^-	-
25.	Formitopsic acid F	9.22	-	527^-	479, 435, 419, 351
	Artist's Bracket				
1.	Ganoderenic acid A	4.38	-	513^-	495, 451, 249
2.	Ganoderenic acid D	4.75	-	511^-	493, 285, 149
3.	Ganoderic acid C6	4.83	-	529^-	481, 467, 437
4.	Ganoderic acid A	4.93	-	515^-	497, 303, 2449
5.	Ganoderic acid H	6.93	-	571^-	511, 467, 437
6.	Ganoderic acid F	7.16	-	569^-	509, 465, 435
