# Supplementary material for: Lipoxygenase and Xanthine Oxidase Inhibition and Antioxidant Potential of Fractions Obtained by Multistep Extraction of Artist’s Bracket (Ganoderma applanatum (Pers.) Pat.) and Red-Belted Bracket (Fomitopsis pinicola (Sw.) P. Karst.)
Source: Antioxidants (Basel). 2026 May 25;15(6):663. doi: 10.3390/antiox15060663 (PMC13295654; doi:10.3390/antiox15060663)
Supplement: Supplementary file 1 [file antioxidants-15-00663-s001.zip › Suppl. S3. Relationship between the content of active compounds and bioactivity (correlation coefficient)..pdf]

Suppl. S3. Relationship between the content of active compounds in the extracts and bioactivity (Pearson correlation coefficients)

	Terpenoids/Sterols		Folin-Ciocalteu reacting substances		Saccharides	
	Artist's bracket	Red-belted bracket	Artist's bracket	Red-belted bracket	Artist's bracket	Red-belted bracket
Ability to inhibit xanthine oxidase activity (LOXI)	0.99	0.97	0.96	0.16	0.23	0.85
Ability to inhibit xanthine oxidase activity (XOI)	0.71	0.97	0.57	0.27	-0.41	0.78
Antiradical properties (ABTS)	0.39	0.33	0.55	0.91	0.96	0.61
Ferric reducing power (RP)	0.69	0.35	0.81	0.77	0.90	0.68
